# Comparative assessment of the bactericidal effect of nanoparticles of copper oxide, silver, and chitosan-silver against *Escherichia coli* infection in broilers

**DOI:** 10.1042/BSR20204091

**Published:** 2021-04-16

**Authors:** Eman I. Hassanen, Eman A. Morsy, Ahmed M. Hussien, Khaled Y. Farroh, Merhan E. Ali

**Affiliations:** 1 Pathology Department, Faculty of Veterinary Medicine, Cairo University, Giza 12613, Cairo, Egypt; 2 Poultry Disease Department, Faculty of Veterinary Medicine, Cairo University, Giza 12613, Cairo, Egypt; 3 Toxicology and Forensic Medicine Department, Faculty of Veterinary Medicine, Cairo University, Giza 12613, Cairo, Egypt; 4 Nanotechnology Department, Agricultural Research Center, Giza 12619, Cairo, Egypt

**Keywords:** Broilers, Escherichia coli, Histopathology, Nanomedicine, nanoparticles

## Abstract

*Escherichia coli* infection is considered one of the most economically important multi-systemic diseases in poultry farms. Several nanoparticles such as silver, chitosan, and copper oxide are known to be highly toxic to several microbes. However, there are no data concerning their success against *in vivo* experimental *E. coli* infection in broilers. Therefore, the present study was designed to investigate the bactericidal effect of low doses of CuO-NPs (5 mg/kg bwt), Ag-NPs (0.5 mg/kg bwt), and Ch-Ag NPs (0.5 mg/kg bwt) against *E. coli* experimental infection in broilers. One hundred chicks were divided into five groups as follows: (1) control; (2) *E. coli* (4 × 10^8^ CFU/ml) challenged; (3) *E. coli* +CuO-NPs; (4) *E. coli* +Ag-NPs; (5) *E. coli* +Ch-Ag NPs. The challenged untreated group, not NPs treated groups, recorded the lowest weight gain as well as the highest bacterial count and lesion score in all examined organs. The highest liver content of silver was observed in Ag-NPs treated group compared with the Ch-Ag NPs treated group. Our results concluded that Ch-Ag NPs not only had the best antibacterial effects but also acted as a growth promoter in broilers without leaving any residues in edible organs. We recommend using Ch-Ag NPs in broiler farms instead of antibiotics or probiotics.

## Introduction

Poultry industry is primarily threatened by numerous microorganisms, which diminish the growth rate and cause broadly financial misfortunes. Among these, *Escherichia coli* is related to different manifestations in broilers and viewed as a significant foodborne pathogen in humans [1]. *E. coli* infection mostly occurs in chickens by using contaminated food, cross-contamination in breeding houses, or through slaughter and handling [2]. Consequently, more accentuation is probably centered on diminishing *E. coli* and other pathogens on poultry farms to reduce contamination with pathogens in prepared meats [3]. To increase livestock productivity, it is important to make diagnosis, treatment, and prevention of diseases. Currently, immunization and numerous antibiotics are used to combat microorganisms, but careless usage of antibiotics may cause wellbeing dangers to consumers [4]. Thus, it is interesting to examine other modern sorts of secure and successful biocidal compounds to battle poultry bacterial infections. Recently, nanotechnology has developed a modern promising innovation for nanoparticle synthesis in the nanometer size, which displays antimicrobial impacts related to their high surface area-to-volume proportion [5]. The antibacterial potentials of different metal and metal oxide nanoparticles such as copper, titanium, zinc, and silver have been well reported [6]. Silver nanoparticles (Ag-NPs) are considered successful antibacterial agents against several microbes such as *E. coli, Vibrio cholera, Salmonella typhi*, and *Pseudomonas aeruginosa* [7,8]. Wide utilization of elemental silver or silver compounds in medical applications is due to the great antimicrobial efficiency of ionic silver (Ag^+^) against various Gram-positive and -negative bacteria as well as fungi in combination of low toxicity against human tissue [9]. Thus, Ag-NPs may be a great candidate as an alternative for the formulation of a modern generation of antibacterial agents used in biological, medical, and pharmaceutical applications [10]. Ag-NPs can create their antibacterial action by disrupting the cell wall and cytoplasm, changing ATP levels, altering permeability and cell membrane respiration, hindering bacterial DNA replication, and creating free radicals, such as reactive oxygen species (ROS) [11]. The toxic effects of Ag-NPs still obscure and *in vivo* information of Ag-NPs toxicities are limited and disputable [12]. A few researchers have found that diverse surface stabilizers have imperative effects on Ag-NPs cytotoxicity. Chitosan is used as an active component of topical wound materials due to its great biocompatibility and antibacterial properties [13]. Numerous investigations have found that chitosan has been considered a good stabilizer for Ag-NPs and its antimicrobial efficacy [14]. In addition, chitosan-coated Ag-NPs showed great efficiency in killing common Gram-positive and -negative microbes, and fungi [15]. Copper oxide nanoparticles have been used in packaging and coatings of food because of their antimicrobial and antifungal properties. Like Ag-NPs, CuO-NPs have a wide spectrum of antibacterial effects against both Gram-positive and -negative microbes [16]. On account of the normal medicines by antibiotics lead to lack of biodiversity, the most punctual utilization of nanoparticles as an antimicrobial agent for treating different microbial infections is being developed [17]. In any case, *in vivo* studies related to the use of nanoparticles against poultry pathogens are excessively limited. Thus, the current study has made an endeavor to discover novel antibacterial agents from metal and metal oxide nanoparticles for treating *E. coli* infections in broiler chickens. The NPs were selected due to their significance in food safety applications.

## Materials and methods

### Bacterial strain isolation, identification, and counting

*E. coli* isolate O_78_ was previously isolated from broiler chicks suffered from high mortalities, serotyped and detected for its virulence via Congo Red binding assay [18]. *E. coli* O_78_ serotype was grown aerobically in nutrient broth at 37°C for 24 h before using as a target organism. The dose was calculated to match 4 × 10^8^ CFU/ml of bacterial isolate.

### Preparation of copper oxide nanoparticles

Copper oxide nanoparticles were prepared by chemical precipitation method according to the method described by **Hassanen et al.** [19].

### Preparation of Ag-NPs

Ag-NPs colloidal solution (17 ± 5 nm) was prepared by co-precipitation protocol through the reduction of silver nitrate (AgNO_3_) (99.99%, Aldrich, U.S.A.) with sodium borohydride (99%, Aldrich, U.S.A.) under boiling conditions [20]. The concentration of silver in the prepared solution was 10 mM.

### Preparation of chitosan-AgNPs

Chitosan-silver nanocomposites (Ch-Ag NCs) were prepared by reduction of silver nitrate by chitosan according to the method described by **Hassanen et al.** [21]. The concentration of silver in the prepared solution was 10 mM.

### Characterization of the prepared nanoparticles

Actual morphology and size of nanoparticles were assessed by High Resolution Transmission Electron Microscope (HR-TEM) operating at an accelerating voltage of 200 kV (Tecnai G2, FEI, Netherlands). Dynamic light scattering (DLS) technique was utilized to estimate the average particle size distribution that was measured by zeta sizer (Malvern, ZS Nano, U.K.). The chemical structure of the prepared nanoparticles was assessed using X-ray diffraction (XRD) technique.

### Acute toxicity study to determine LD_50_ of the selected nanoparticles

A total of 60 commercial broiler (Cobb 500) chicks (7-day-old) were used to determine LD_50_ of Ag-NPs, Ch-Ag NPs, and CuO-NPs. They were divided into different groups according to different doses of the treated nanoparticles. The LD_50_ value was calculated according to **Weil** [22] as in the following equation:

$$\mathrm{Lo}g\;LD_{50}\;=\;Log\;D_{a}\;+\;d\;(f\;+\;1).$$

**Log Da** = log of the lowest of the four dosage levels used, **d** = logarithm of geometric factor (R), **f** = R-values in the table.

### Animals and experimental design

All the procedures of the experiment were done according to the guidelines of the Institutional Animal Care and Use Committee at Cairo University and approved by Vet-CU-IACUC (approval number: 0722019057), Cairo, Egypt.

A total of 100 one-day-old mix (Cobb 500) broiler chicks were obtained from El-Hawamdya-Giza. The birds were resided in pens on straw litter and reared in standard hygiene conditions in a building with regulated temperature and humidity. The birds had permanent access to drinking water and received *ad libitum* complete feed mixtures appropriate for the rearing period according to the nutrient requirements of broilers.

The chicks were randomly divided into five groups of 20 birds each. Group (1) was kept as a control group and received normal saline daily by oral gavage for 7 days (control negative). Group (2) was challenged at 7th day of age by crop gavages with 4 × 10^8^ CFU/ml/bird of *E. coli* serogroup O_78_ in PBS for 2 successive days according to the method described by **Awaad et al.** [23] and left untreated; group (3) was challenged and treated by 5 mg/kg bwt CuO-NPs daily by oral gavage for 7 days; group (4) was challenged and treated by 0.5 mg/kg bwt Ag-NPs daily by oral gavage for 7 days; group (5) was challenged and treated by 0.5 mg/kg bwt Ch-Ag NPs daily by oral gavage for 7 days. The dose of different nanoparticles is obtained from the acute toxicity study which represented 1/20 LD_50_. All birds were monitored and weighted weekly all over the experimental period.

### Sampling

At 21 days, all birds were admitted to PM room to collect blood and organ samples. Blood samples were collected aseptically from wing vein and used freshly for bacterial count. All birds were slaughtered by exsanguination without using anesthesia to collect liver, spleen, intestine, kidneys, heart, and bursa of Fabricius. Some of these organs preserved at −80ºC till used for bacterial re-isolation, while others preserved in 10% neutral buffered formalin for histopathological examinations.

### Bacterial re-isolation

The blood and organ (liver and spleen) tissue homogenates were then ten-fold serially diluted before platting on EMB agar for plate counting. Biochemical identification was performed using API 20-Etest kit (bioMérieux Inc., Marcy l’Etoile, France) according to the manufacturer's instructions [24]. Serological identification of the isolated *E. coli* was done using *E. coli* antisera (Denka Seiken, Japan) according to the method described by **Blanco et al.** [25].

### Histopathological examinations

Formalin-fixed tissue specimens were processed via conventional methods and sliced into 4 mm sections to obtain paraffin-embedded tissue sections stained by H&E to be examined under light microscope for histopathological examination [26].

Microscopic grading and scoring were performed to document lesion severity of the examined organs in different treated groups. The grading criteria for degenerative, necrotizing, and inflammatory lesions were assessed as none, slight, mild, moderate, and severe as follow; 0 (normal histology), 1 (<25%), 2 (25:50%), 3 (50:75%), 4 (>75% tissue damage) according to method described by **Hassanen et al.** [27]. While grading scheme for multifocal lesions assessed according to the method documented by **Hassanen et al.** [28] as follow: (0) no foci; (1) <3 foci; (2) 3–6 foci; (3) 7–12 foci; (4) >12 foci.

### Nanoparticle content in different organs

Flame atomic absorption spectrophotometer (ZEISS, AAS5, and Germany) was used to measure the contents of copper and silver in muscle and some edible organs such as heart, liver, and spleen tissue homogenate [19]. Briefly, concentrated nitric acid and 30% H_2_O_2_ were added to 0.5 g tissue samples and kept overnight, then heated in a microwave digestion system (ETHOS One; Milestone, Sorisole, Italy) till it became completely digested and colorless. Afterward, the samples were allowed to cool, and the remaining solutions were diluted with 2% nitric acid.

### Statistical analysis

Statistical analysis was performed utilizing SPSS version 16.0 software (SPSS Inc., Chicago, IL, U.S.A.). Values were expressed as means ± SEM. Comparison of means between several groups was performed by one-way analysis of variance (ANOVA) and independent *t* test was used to compare between two groups. Values were considered statistically significant at *P*≤0.05.

## Results

### Characterization of nanoparticles

**Figure 1:**
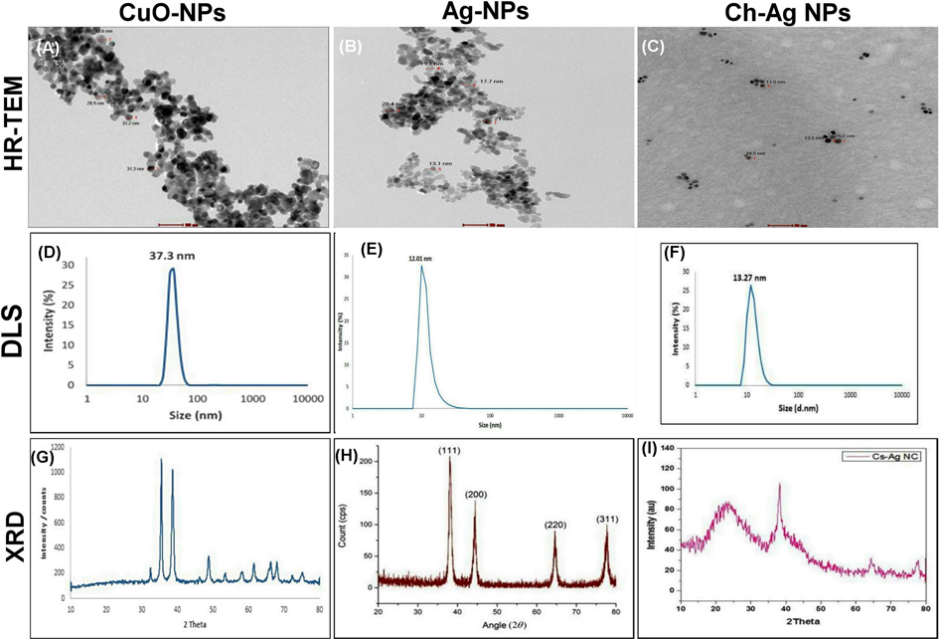
Characterization of the prepared nanoparticles (**A–C**) High resolution-transmission electron microscopic image of different NPs. (**D–F**) Particle size distribution curve obtained from DLS measurements. (**G–I**) XRD pattern of NPs.

HR-TEM images showed spherical-shaped CuO-NPs with average size ∼28.9–45.6 nm (Figure 1A). Ag-NPs showed well uniformed spheres with average size of 17 ± 5 nm (Figure 1B). Ch-Ag NCs showed spherical shaped nanoparticles with average particle size 17.5 nm distributed homogeneously in the Ch matrix (Figure 1C). The particle size distribution curve obtained from DLS measurements were 37.3, 17.3, 20 nm for CuO-NPs, Ag-NPs, and Ch-Ag NCs, respectively (Figure 1D–F). XRD pattern of CuO-NPs showed peaks at 2θ = 32.48°, 35.54°, 38.64°, 48.85°, 61.52°, 65.66, 66.34, and 68.02° were assigned to (110), (−111), (111), (−202), (−113), (022), (−311), and (220) of CuO nanoparticles, indicating that the crystalline structure of synthesized Cu nanoparticles presented a hexagonal wurtzite structure (Zincite, JCPDS 04-005-4712) (Figure 1G). XRD pattern of Ag-NPs showed sharp intense and narrow peaks at 38.14°, 44.41°, 64.61°, and 77.74° 2θ angles those corresponding to *hkl* parameters of (111), (200), (220), and (311), respectively (Figure 1H). The obtained diffraction pattern was compared with the standard ICCD library installed in PDF4 software, card no: (04-003-5625). XRD pattern of Ch-Ag NCs showed a peak of chitosan at 2θ value of the broad peak ∼15–35°. The peaks of silver were indexed to the face-centered cubic structure which is in good agreement to the JCPDS card No. 04-004-8730. The three silver peaks obtained belong to the (111), (220), and (311) reflections, respectively. The results showed that the synthesized nanoparticles were Ag-NPs because the position and relative intensity of all the diffraction peaks of the samples were consistent with the crystalline pattern of silver. The presence of chitosan, silver, and the absence of impurity phases were evident from the XRD image (Figure 1I).

### Acute toxicity study of the prepared nanoparticles

Mortalities recorded within 24 h after oral administration of CuO-NPs, Ag-NPs, and Ch-Ag NPs in different treatment groups were recorded in (Table 1). The calculated oral LD_50_ of CuO-NPs, Ag-NPs and Ch-Ag NPs in 7-day-old chicks (Cobb 500) equals 100, 10, 381 mg/kg bwt for each nanoparticle, respectively.

**Table 1:** Mortality data in 7-day-old chicks received single oral doses of different nanoparticles in different groups

Groups	1	2	3	4
CuO-NPs				
Dose (mg/kg)	27	40.5	60.75	91.125
Number of birds/group	5	5	5	5
Number of dead birds/group	0	1	2	5
Ag-NPs				
Dose (mg/kg)	3.1	6.2	12.4	24.8
Number of birds/group	5	5	5	5
Number of dead birds/group	0	1	3	5
Ch-Ag NPs				
Dose (mg/kg)	120	240	480	960
Number of birds/group	5	5	5	5
Number of dead birds/group	0	2	2	5

### The effect of different treatments on the body weight and mortality of birds

**Figure 2:**
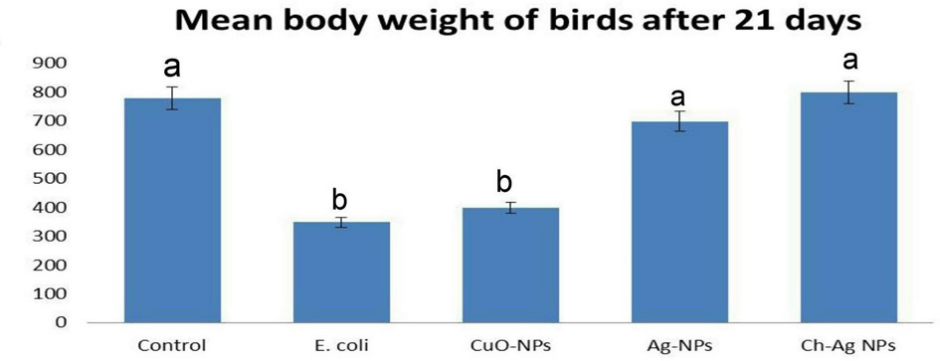
The effect of different treatments on mean body weights of birds Bar charts representing mean body weights of birds in different groups. Values were presented as mean ± SEM (*n* =20 birds/ group). Values with different letters (a,b) are considered significantly different at *P*≤0.05.

The results summarized in Figure 2 showed remarkable reduction in mean body weight of broiler chickens in challenged untreated group. There was a noticeable increasing in body weights of chickens in group treated with Ch-Ag NCs compared with control group. On the other hand, there was no significant difference in mean body weight of birds in nanoparticles treated groups compared with control group. The highest mortality rate was recorded in the challenged untreated group (40%) followed by CuO-NPs treated group (20%). On the other side, no mortality recorded in both control group and those treated with either Ag-NPs or Ch-Ag NPs.

### Bacterial re-isolation

There was a remarkable reduction in the *E. coli* count in groups treated either with Ag-NPs or Ch-Ag NPs (∼80, 95%, respectively) in blood and different organs (liver and spleen) compared with challenged untreated group. Approx. 50% reduction in the *E. coli* count observed in both blood and organs obtained from CuO-NPs treated group compared with the challenged untreated group (Table 2).

**Table 2:** The effect of different treatments on the mean *E. coli* count (CFU/ml) in the blood and different organs after 21 days post challenge

	Blood	Liver	Spleen
**Control**	0 ± 0^A^	0 ± 0^A^	0 ± 0^A^
***E. coli***	180 ± 42^B^	219 ± 54^B^	197 ± 46^B^
**CuO-NPs**	115 ± 12^B,b^	150 ± 56^B,b^	130 ± 22^B,b^
**Ag-NPs**	35 ± 5^C,c^	44 ± 7.5^C,c^	40 ± 2.2^C,c^
**Ch-Ag NPs**	9 ± 1.1^C,a^	8.5 ± 2^C,a^	9.8 ± 1.2^C,a^

Value was demonstrated as mean ± SEM. Value with different letters considered significantly different at *P*≤0.05 (*n*=7 birds/group). Capital letters indicate significance between all five groups while small letters indicate significance between the three nanoparticles treated groups.

### Nanoparticles contents in muscle and edible organs

There was a significant increase in copper contents in muscle, spleen, and heart in CuO-NPs treated group compared with the control group (Table 3). Whereas, a significant increase in silver contents of liver of birds in Ag-NPs treated group were reported. On the other hand, there were no significant difference in silver content in muscle, spleen, and heart in the groups treated with either Ag-NPs or Ch-Ag NPs compared with the control group (Table 4).

**Table 3:** Copper contents in different organs of CuO-NPs treated or untreated groups

	Control	CuO-NPs
**Muscle**	1.7 ± 0.02^a^	2.2 ± 0.07^b^
**Liver**	2.5 ± 0.12^a^	2.6 ± 0.32^a^
**Spleen**	2.5 ± 0.23^a^	2.6 ± 0.13^a^
**Heart**	1.5 ± 0.21^a^	2.2 ± 0.11^b^

Values were presented as mean ± SEM (*n*=7 birds/group). Values with different letters considered significantly different at *P*≤0.05.

**Table 4:** Silver contents in different organs

	Control	Ag-NPs	Ch-Ag NPs
**Muscle**	0 ± 0^A^	0.001 ± 0^Aa^	0 ± 0^A,a^
**Liver**	0 ± 0^A^	31.3 ± 1.24^B,b^	3.4 ± 0.26^A,c^
**Spleen**	0 ± 0^A^	4.2 ± 0.9^B,b^	0.001 ± 0^A,c^
**Heart**	0 ± 0^A^	0.002 ± 0^A,a^	0 ± 0^A,a^

Values were presented as mean ± SEM (*n*=7 birds/ group). Values with different letters considered significantly different at *P*≤0.05. Capital letters A and B indicate significance between all groups while small letters a, b, and c indicate significance between nanoparticles treated groups.

### Histopathological examinations

Microscopic pictures in all examined organs obtained from the control negative group showed normal histological structures. On the other hand, the *E. coli* challenged group showed severe to moderate pathological alterations in all examined organs with remarkable improvements in all the treated groups.

**Figure 3:**
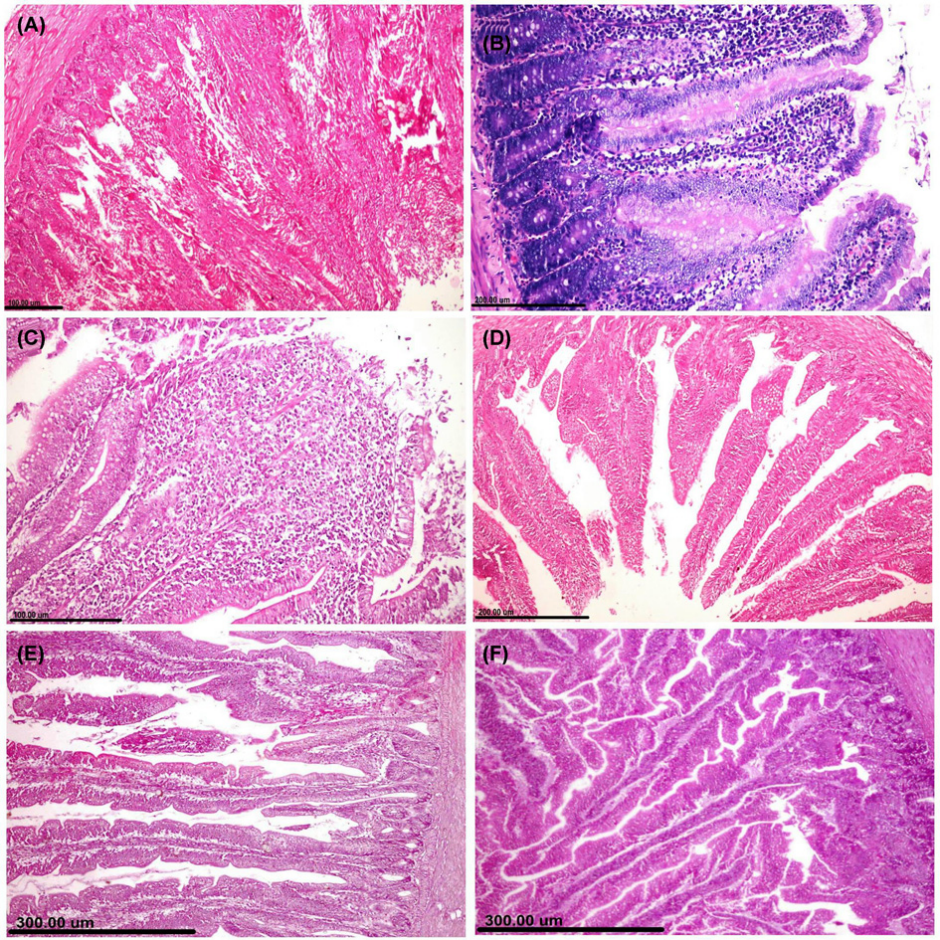
The effect of different treatments on the histopathological pictures of the intestine Photomicrographs of intestinal tissue sections stained with H&E representing, (**A–C**) *E. coli* challenged untreated group showing. (A) Complete destruction of the intestinal villi with fibrinous adhesion. (B) Necrosis of intestinal epithelium with extensive hyperplasia of goblet cells. (C) Extensive widening of lamina propria with inflammatory cells and exudates. (**D**) CuO-NPs treated group showing shortening of intestinal villi with reduction of crypt depth. (**E**) Ag-NPs treated group showing increasing in both height of intestinal villi and crypt depth with minimum inflammatory reactions. (**F**) Ch-Ag NPs treated group showing remarkable increasing in the intestinal villi and crypt depth.

**Small intestine** of the challenged group showed extensive acute enteritis with destruction of intestinal villi (Figure 3A). There were extensive degenerations and necrosis in the epithelial lining intestinal mucosa associated with remarkable hyperplasia of the goblet cell (Figure 3B). Fibrinous exudates, necrotic cell debris, and inflammatory cells were collected and forming pseudo-membrane covering the intestinal mucosa. Lamina propria and submucosa severely infiltrated with heterophils and mononuclear inflammatory cells (Figure 3C). Remarkable improvements were recorded in all the treated groups, but the best microscopic picture observed in groups treated with Ch-Ag NPs. Otherwise, the group treated with CuO-NPs showed degeneration and necrosis in some of the epithelial lining intestinal mucosa associated with mild to moderate inflammatory cells infiltration in lamina propria and submucosa (Figure 3D). In spite of extensive hyperplasia of goblet cells and mild inflammatory reactions observed within the intestinal mucosal layers of AgNP-treated group (Figure 3E), Ch-Ag NPs treated group showed normal histological structures (Figure 3F).

**Figure 4:**
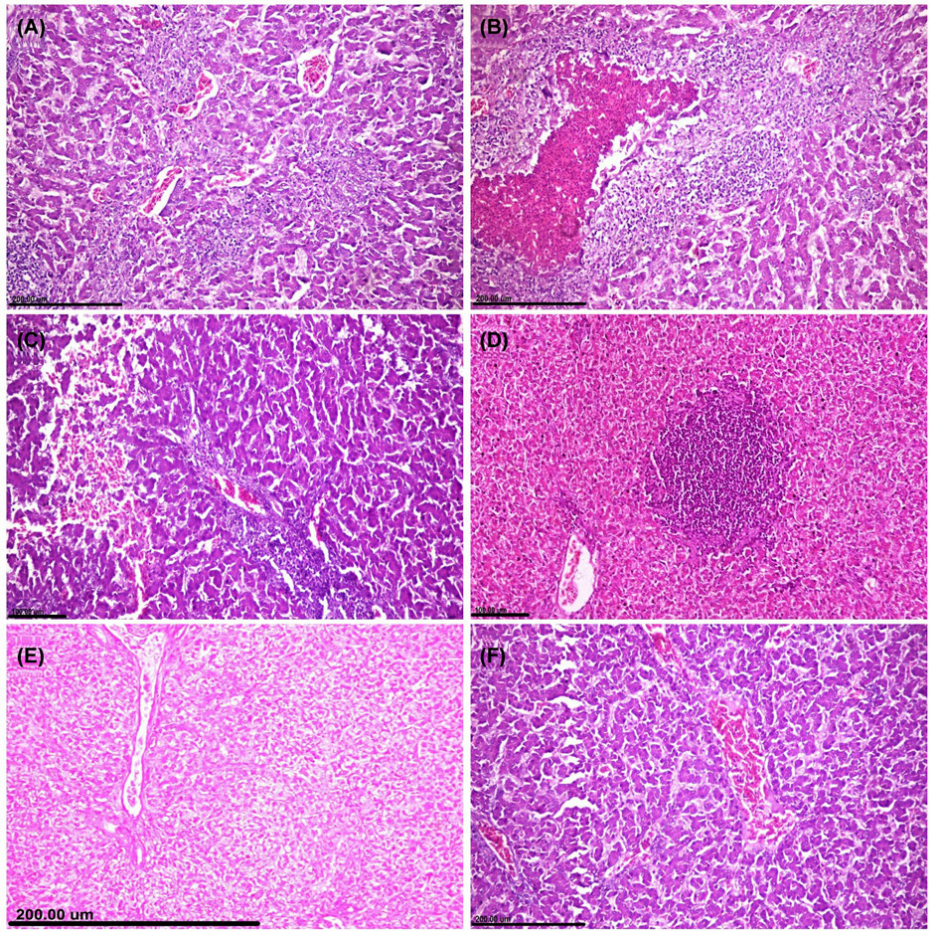
The effect of different treatments on the histopathological pictures of the liver Photomicrographs of hepatic tissue sections stained with H&E representing, (**A–C**) *E. coli* challenged untreated group showing. (A) Coalescent areas of hepatocellular coagulative necrosis infiltrated with mononuclear inflammatory cells. (B) Portal tried showing severe congestion and inflammatory cells infiltrations. (C) Focal hemorrhage. (**D**) CuO-NPs treated group showing focal coagulative necrosis infiltrated with inflammatory cells. (**E**) Ag-NPs treated group showing diffuse hepatocellular cytoplasmic vacuolization. (**F**) Ch-Ag NPs treated group showing normal histological structures.

**Liver** of the challenged group showed extensive hepatocellular degeneration and necrosis. Multifocal areas of hepatocellular coagulative necrosis were detected and infiltrated with inflammatory cells replacing the hepatic parenchyma (Figure 4A). Cholangiohepatitis noticed in some cases and characterized by portal edema and inflammatory cells infiltration (Figure 4B). There was hyperplasia in the epithelial lining bile duct associated with the presence of newly formed bile ductules. Perihepatitis observed in most sections manifested by extensive widening of the hepatic capsule by fibrinous exudates and inflammatory cells infiltrations. Multifocal to coalescent areas of hemorrhage were noticed within the hepatic parenchyma (Figure 4C). Group treated with CuO-NPs showed focal area of hepatocellular necrosis infiltrated with inflammatory cells (Figure 4D). Group treated with Ag-NPs showed moderate to diffuse hepatocellular cytoplasmic vacuolization (Figure 4E). Remarkable improvements were recorded in the group treated with Ch-Ag NPs and the liver sections appeared with normal histological structures (Figure 4F).

**Figure 5:**
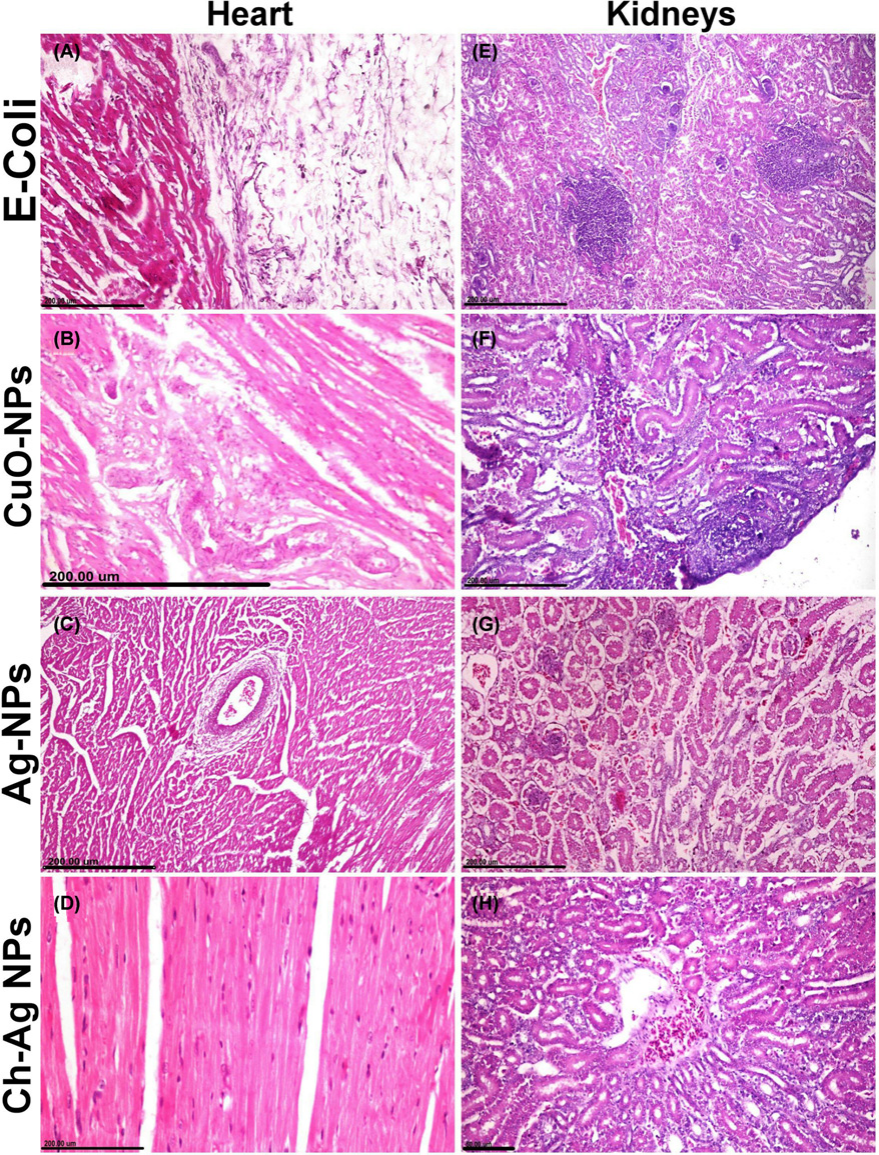
The effect of different treatments on the histopathological pictures of the heart and kidney tissues (**A–D**) Photomicrographs of cardiac tissue sections stained with H&E representing, (A) *E. coli* challenged untreated group showing fibrinous pericarditis. (B) CuO-NPs treated group showing coagulative necrosis and vacuolar degeneration of cardiac muscle. (C,D) Groups treated with Ag-NPs and Ch-Ag NPs respectively showing normal histological structures. (**E–H**) Photomicrographs of kidney sections stained with H&E representing, (E) *E. coli* challenged untreated group showing focal interstitial nephritis. (F) CuO-NPs treated group showing moderate tubulointerstitial nephrotoxic nephritis. (G) Ag-NPs treated group showing marked interstitial edema and hemorrhage with minimum inflammatory cells infiltrations. (H) Ch-Ag NPs treated group showing normal histological structures.

**Heart** of the challenged group showed moderate fibrinous pericarditis characterized by congestion of blood vessels, thickening in the pericardium by fibrinous exudates and inflammatory cell infiltrations (Figure 5A). Cardiac muscle noticed severe degeneration and necrosis associated with congestion and inflammatory cell infiltration. CuO-NPs treated group showed mild to moderate degeneration and necrosis in the cardiac muscle (Figure 5B). On the other side, groups treated with either Ag-NPs (Figure 5C) or Ch-Ag NPs (Figure 5D) showed remarkable improvements and the organ appeared with normal histological structures.

**Kidneys** of the challenged group showed interstitial tubule-nephritis characterized by interstitial congestion, hemorrhage, edema, and inflammatory cells infiltration (Figure 5E). Renal tubular epithelial cells suffered from several degenerative changes and necrosis with intracellular and intraluminal hyaline cast and droplets. Kidneys of the group treated with CuO-NPs showed moderate degeneration and necrosis in the epithelial lining renal tubules associated with interstitial inflammatory reactions (Figure 5F). Ag-NPs treated group showed several degenerative changes in the tubular epithelium with interstitial hemorrhage (Figure 5G). Remarkable improvements observed in the group treated with Ch-Ag NPs (Figure 5H) compared with other nanoparticles-treated group and the kidneys appeared with normal histological structures.

**Figure 6:**
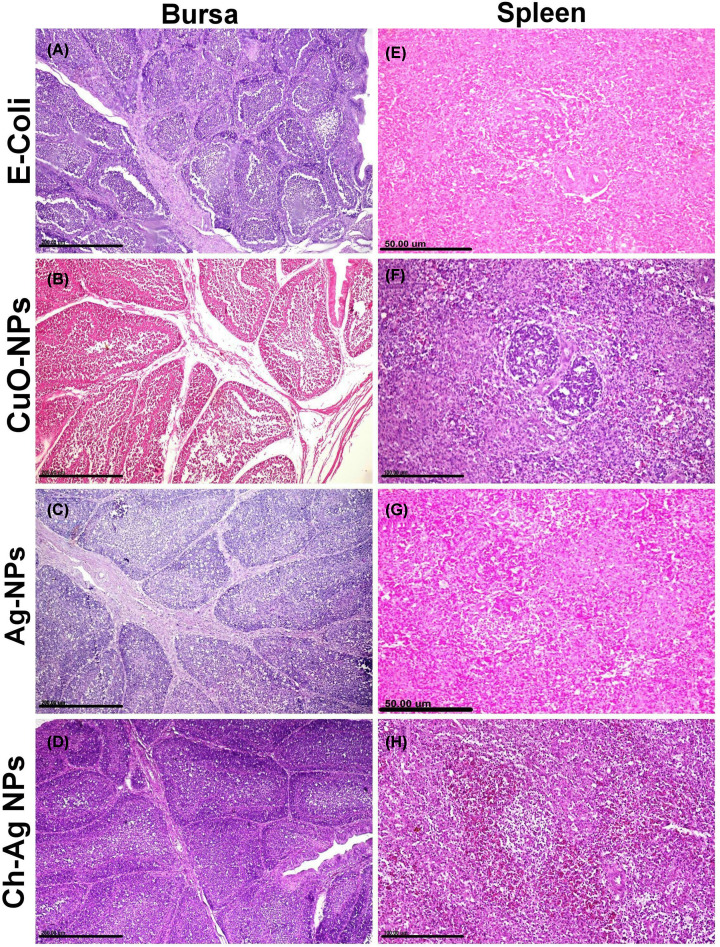
The effect of different treatments on the histopathological pictures of bursa of Fabricius and spleen (**A–D**) Photomicrographs of bursa tissue sections stained with H&E representing, (A) *E. coli* challenged untreated group showing widening of interfollicular septa by fibrinous exudates and inflammatory cells with marked lymphocytic depletion. (B) CuO-NPs treated group showing moderate lymphocytolysis with prominent basement membrane between follicular cortex and medulla. (C) Ag-NPs treated group showing extensive lymphocytolysis with interfollicular fibrosis and inflammatory cells infiltrations. (D) Ch-Ag NPs treated group showing normal histological structures. (**E–H**) Photomicrographs of spleen tissue sections stained with H&E representing, (E) *E. coli* challenged untreated group showing mild lymphocytic cell depletion in periarteriolar lymphoid follicles of splenic white pulp. (F) CuO-NPs treated group showing extensive lymphoid cell depletion in lymphoid follicles with huge number of tangible body macrophages. (G,H) Groups treated with Ag-NPs and Ch-Ag NPs, respectively, and showing normal histological structures.

**Spleen** of the challenged group showed mild to moderate pathological alterations manifested by lymphocytic cell depletion in some lymphoid follicles (Figure 6A). On the other hand, spleen tissue sections in nanoparticle-treated groups showed normal histological structures (Figure 6B–D).

**Bursa of Fabricius** of the challenged group showed extensive lymphocytic cell depletion in most lymphoid follicles associated with marked thickening in the inter follicular septa by edema and inflammatory cell infiltrations (Figure 6E). Group treated with CuO-NPs showed moderate lymphocytolysis with prominent follicular septa between cortex and medulla (Figure 6F). In spite of moderate lymphocytolysis occurred in some lymphoid follicles of Ag-NPs treated group (Figure 6G), bursa obtained from Ch-Ag NPs treated group showed normal histological structures (Figure 6H).

**Figure 7:**
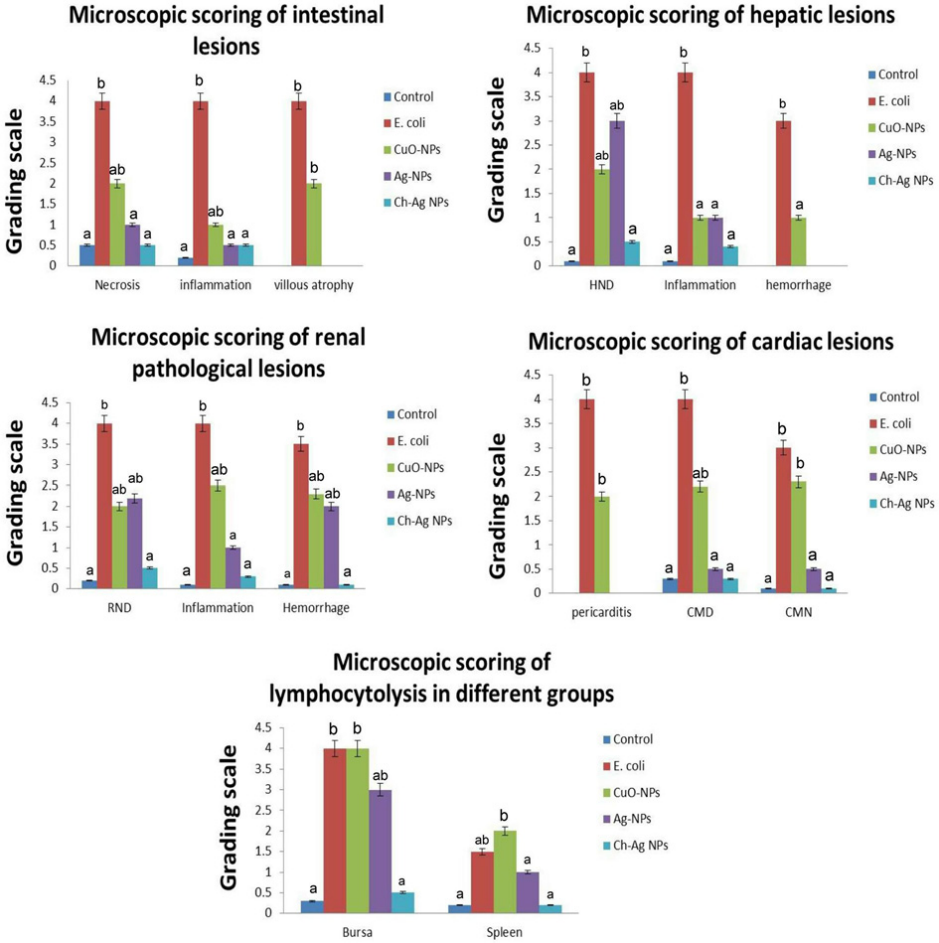
Bar charts representing microscopic lesion scoring in different organs of different treated groups Values were presented as mean ± SEM (*n*=7 birds/ group). Values with different letters considered significantly different at *P*≤0.05. Abbreviations: CMD, cardiac muscle degeneration; CMN, cardiac muscle necrosis; HND, hepatocellular necrosis and degeneration; RND, renal tubular necrosis and degeneration.

Lesion scoring in all examined organs of different treated groups was illustrated in (Figure 7). The highest score noticed in the *E. coli* challenged group, while the lowest score noticed in the group treated with Ch-Ag NPs. Reduction in lesion scoring observed in an ascending order for CuO-NPs and Ag-NPs.

## Discussion

The expansion of bacterial strains showing resistance from different antimicrobials has encouraged researchers just as the food business administrators to search for other antimicrobial options. Nanotechnology may introduce a probable solution for this problem and there are different kinds of nanoparticles usually viewed as a wide-range antimicrobial specialist especially metal oxide and Ag-NPs [29,30]. The antibacterial potentials of NPs are poorly understood in broiler chickens; therefore, our study was designed to investigate and compare between the possible antibacterial effect of nanoparticles of CuO, Ag, and Ch-Ag against *E. coli* experimental infections in broiler chickens.

In the present study, *E. coli* challenged, and untreated groups showed extensive reduction in body weight and this is consistent with **Rosa et al.**, who attributed the decreased weight gain to the oxidative stress caused by *E. coli* [31]. These results reflected on the pathological pictures and bacterial re-isolation in different organs of this group, which showed the highest lesion score and bacterial count in all examined organs. Our histopathological results were come in accordance with **Sawah et al.**, who observed fibrinonecrotic enteritis, fibrinous perihepatitis, and fibrinous pericarditis in chickens infected with *E. coli* [32]. Another recent study reported that *E. coli* infection resulted in depletion of lymphocytes in bursa and spleen [33].

The present study showed an increase in the bactericidal effect in an ascending order for CuO-NPs, Ag-NPs, and Ch-Ag NPs. Although the CuO-NPs exert moderate antibacterial effects against *E. coli* experimental infection in broiler chickens, it is considered to be toxic to the broilers and elicit pathological alterations in all examined organs. Flame atomic absorption spectroscopic results observed an increase in the copper content in muscle and other edible organs, suggesting that the microscopic lesions observed in the CuO-NPs treated group is related to the CuO-NPs itself not to *E. coli* infections. Indeed, CuO-NPs reduced ∼50% of the bacterial count in the liver, spleen, and heart compared with the challenged untreated group. Our findings were some extent similar to **Al-Jassani and Raheem**, who found that CuO-NPs have considerable powerful inhibitory and anti-bacterial effect on *E. coli* [34]. Several studies have investigated the in vitro antimicrobial effects of CuO-NPs on different kinds of food-borne pathogens as *Staphylococcus aureus, E. coli*, and *Klebsiella pneumonia* [35]. The antibacterial activity of CuO-NPs could be attributed to the sudden decline in bacterial cell membrane integrity in addition to the release of ROS, which contribute to the degradation of several biomolecules that affect also on normal cell viability [36,37].

Rather than CuO-NPs treated group, other NP-treated groups showed marked improvements in body weights and the best results were observed in the group treated with Ch-Ag NPs. Our findings may be related to the biological effects of both silver and chitosan on intestinal harmful bacteria, which resulted in improved growth as the absorption of nutrients was increased [38]. Additionally, NPs could increase intestinal absorption and utilization of minerals required for improving growth performance by increasing the surface area [39]. Our histopathological results showed an increase in the height of the intestinal villi and crypt depth in both Ag and Ch-Ag NPs treated groups, suggesting improved mineral and nutrient absorption in such groups. Similarly, as antibiotics, Ag-NPs are relied upon improving the well-being of animals. That is, Ag-NPs allowing them the chance to consume fewer supplements on the metabolic exertion required for immunological control and to use additional supplements for other physiological and gainful purposes [40].

The best antibacterial effect was noticed in Ch-Ag NPs treated group compared with other NPs treated groups, which manifested by a marked reduction in both bacterial count and lesion score in all examined organs. This is attributed to the potent antibacterial capacity of both silver and chitosan nanoparticles [41,42]. The bactericidal action of Ag-NPs might be because of a brief balance of the surface electric charge of the bacterial membrane promoting bacterial death [43–45]. Moreover, the generation of ROS restrains the antioxidant defense mechanism leading to further damage to the cell membrane. Chitosan is a nonpoisonous biopolymer obtained from shellfish and showed an amazing antibacterial activity [46]. **Du et al.** found that the antimicrobial properties of chitosan were improved extensively by loading it with different metals [47]. The bactericidal effects of NPs depend on their particular physicochemical properties [48,49]. Rather than traditional antibiotics, NPs have peculiar dimensions <100 nm. Their uniquely small size outcomes in novel properties, as more prominent interaction with cells because of a larger surface area-to-mass ratio and flexible and controllable application [50,51].

The current study revealed a significant increase in silver content of the liver of birds in the group treated with Ag-NPs in contrast with those treated with Ch-Ag NPs. Our findings suggest that the accumulation of Ag-NPs in the liver of broiler chickens may be transferred to consumers leading to several side effects. These results are in harmony with several previous studies showed a marked increase in Ag retention in the liver more than in muscular tissue and other organs in broiler chickens [52,53]. Chitosan nanoparticles contain unique functional groups (amino groups) that interact with silver ions, in addition, nanoparticles as well act as capping sites for nanoparticle stabilization [54]. Moreover, Ch-NPs not only act as matrix or capping agent but also act as stabilizing agent for Ag-NPs by forming a network on NP surface allows the homogeneous Ag-NPs distribution on the surface, with no visible aggregation effects [55]. This, in turn, determines the potential bactericidal effects of Ch-Ag NPs and their bioavailability by covering the external surface of the carrier [56]. We suggest that coating of silver NP core by chitosan can reduce particle aggregation and improve their solubility, bioavailability, and stability, so that, it prevents accumulation of silver in organs and increasing their excretion.

## Conclusions

From our results, we concluded that Ch-Ag NPs had a powerful bactericidal activity against *E. coli*, which neither reduced body weight gains nor leaving toxic residues in muscles and edible organs. Alternatively, CuO-NPs not only reduced body weights of birds but also caused extensive pathological alterations in different organs associated with increasing copper levels in such organs. Our results find that chitosan nanoparticles not only have the ability to increase the antibacterial effect of Ag-NPs but also it can reduce their bioaggregation and toxicity in different organs. Therefore, we highly recommended using Ch-Ag NPs as an alternative antibacterial agent in treating infections without taking the risk of developing resistant bacterial strains as with antibiotics. In addition, further studies are required to discuss the mechanism of action of chitosan nanoparticles and how it can prevent the accumulation of silver or other NPs in body organs. Moreover, more studies are needed to compare the effect of metal nanoparticles and those coating with chitosan to confirm the ability of Ch-NPs either on detoxifying or improve the efficacy of many metallic and metal oxide nanoparticles.

## Data Availability

All data will be available on request to the corresponding author.

## Abbreviations

Ag-NP: silver nanoparticle
bwt: body weight
Ch-AgNC: chitosan-silver nanocomposite
DLS: dynamic light scattering
H&E: hematoxylin and eosin
HR-TEM: High Resolution Transmission Electron Microscope
PM: postmortem examinations
ROS: reactive oxygen species
XRD: X-ray diffraction
