# Correction: Comparative assessment of the bactericidal effect of nanoparticles of copper oxide, silver, and chitosan-silver against *Escherichia coli* infection in broilers

**DOI:** 10.1042/BSR20204091_COR

**Published:** 2025-03-31

**Keywords:** broilers, *Escherichia coli*, histopathology, nanomedicine, nanoparticles

The authors of the original article titled ‘Comparative assessment of the bactericidal effect of nanoparticles of copper oxide, silver, and chitosan-silver against *Escherichia coli* infection in broilers’ (DOI: https://doi.org/10.1042/BSR20204091) would like to correct the affiliation of the author Khaled Yehia Farroh.

The original affiliation: Nanotechnology Department, Agricultural Research Center, Giza 12619, Cairo, Egypt.

Should instead be: Nanotechnology and Advanced Materials Central Lab., Agricultural Research Center, Giza, Egypt. Regional Center for Food and Feed, Agricultural Research Center, Giza, Egypt

